# Lumbar laminotomy and replantation for the treatment of lumbar spinal epidural lipomatosis: A case report

**DOI:** 10.1097/MD.0000000000026795

**Published:** 2021-07-30

**Authors:** Keshi Yang, Changbin Ji, Dawei Luo, Kunpeng Li, Hui Xu

**Affiliations:** Department of Orthopaedics, Liaocheng People's Hospital, Liaocheng City, Shandong Province, China.

**Keywords:** lumbar laminotomy and replantation, spinal epidural lipomatosis, surgical decompression

## Abstract

**Rationale:**

Lumbar spinal epidural lipomatosis (SEL) is a rare condition characterized by excessive overgrowth of extradural fat within the lumbar spinal canal. Surgical decompression is commonly performed to treat symptomatic SELs. Fenestration or laminectomy with epidural fat debulking was a routine surgical procedure according to the literature, that may be causing postoperative lumbar instability. In the present study, we presented a brief report of lumbar SEL and introduced another surgical approach.

**Patient concerns:**

A 55-year-old man complained of severe low back pain and right leg radicular pain for a year, accompanied by neurogenic intermittent claudication. He received a variety of conservative treatments, including non-steroidal anti-inflammatory drugs, acupuncture, and physical therapy. However, his pain did not diminish. Finally, a posterior epidural mass in the dorsal spine extending from the L3 to L5 level, which caused dural sac compression was found on lumbar magnetic resonance imaging. This mass was homogeneously hyperintense in both T1W1 and T2W1 images, suggestive of epidural fat accumulation.

**Diagnoses:**

Lumbar SEL.

**Interventions:**

The patient underwent lumbar laminectomy, epidural fat debulking, and spinous process-vertebral plate in situ replantations.

**Outcomes:**

The patient presented with complete recovery of radiculopathy and low back pain after surgery. Postoperative magnetic resonance imaging showed that the increased adipose tissue disappeared, and the dural sac compression was relieved. A computed tomography scan revealed the lumbar lamina in situ. He was able to walk normally and remained relatively asymptomatic for 12 months after the operation at the last follow-up visit.

**Lessons:**

Lumbar laminotomy and replantation provide an ideal option to treat lumbar SEL because it can achieve sufficient and effective decompression, simultaneously reconstruct the anatomy of the spinal canal, and reduce the risk of iatrogenic lumbar instability.

## 1 Introduction

Spinal epidural lipomatosis (SEL), a rare space-occupying lesion in the spinal canal, is defined as the diffuse overgrowth of epidural adipose tissue causing spinal canal compression and neurologic deficits.^[1,2]^ The symptoms are similar to those caused by lumbar disc herniation or lumbar spinal stenosis, such as lower back pain, numbness, pain, weakness, and cauda equina syndrome.^[3]^ Surgical interventions will be suggested when patients exhibit acute neurological deterioration or when conservative treatment is ineffective. Fenestration or laminectomy with epidural fat debulking is a routine surgical procedure according to the literature,^[4,5]^ which damages the structure of the posterior spine and affects stability. In the current report, we present a case of lumbar SEL (L3–L5); lamina osteotomy and replantation in situ were performed, and the patient showed significant improvement in neurological symptoms and low back pain postoperatively.

## 2 Case summary

A 55-year-old man (weight, 70 kg; height, 170 cm; and body mass index, 24.20 kg/m^2^) presented with a 1-year history of low back pain and right leg radicular pain, with neurogenic intermittent claudication. The patient reported a pain score of 8 on the visual analog scale score (0 = no pain and 10 = extremely painful), and the Oswestry Disability Index was 78%. He denied a history of steroid use and a history of endocrine and metabolic diseases.

Physical examination revealed numbness in the skin of the left calf and sole of the left foot, accompanied by a decrease in touch and pinprick sensation in the same area. There was no significant decrease in muscle strength of both lower limbs. Bilateral knee tendon reflexes and Achilles tendon reflexes were decreased.

**Figure 1:**
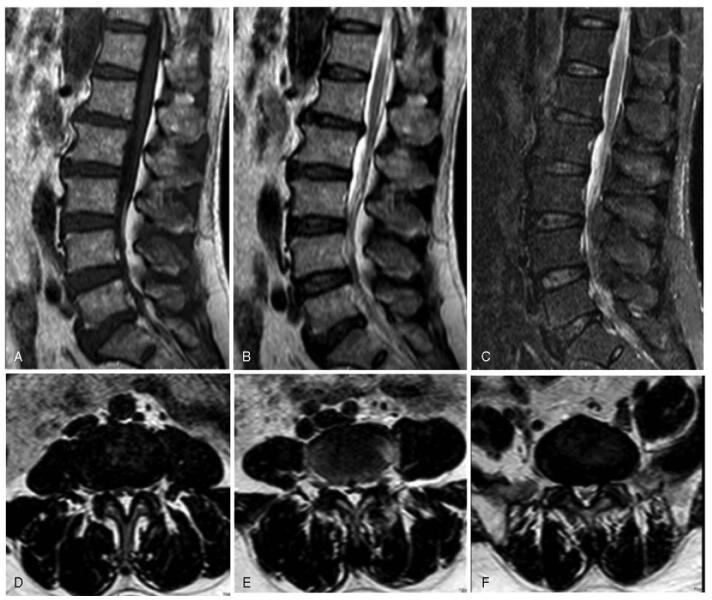
Lumbar MRI showed that the adipose tissue compresses the thecal sac at L3–S1 levels. (A) T1-weighted sagittal image, (B) T2-weighted sagittal image, (C) fat-suppressed T2-weighted image, (D) lumbar axial MRI (L3–L4 level), (E) lumbar axial MRI (L4–L5 level), and (F) lumbar axial MRI (L5–S1 level). MRI = magnetic resonance imaging.

Before hospitalization, the patient received a variety of conservative treatments, including non-steroidal anti-inflammatory drugs, acupuncture, and physical therapy. However, his symptoms did not alleviate. Laboratory tests showed that serum corticosteroid levels, thyroid hormone levels, serum glucose level, and serum lipid profiles of the patient were within normal limits. Magnetic resonance imaging (MRI) of the lumbar spine showed a posterior epidural mass in the dorsal spine extending from the L3 to L5 level, causing dural sac compression. This mass was homogeneously hyperintense in both T1W1 and T2W1 images, suggestive of epidural fat accumulation (Fig. 1). According to the MRI grading by Borré et al,^[6]^ the current patient was classified as grade III, with severe overgrowth of epidural fat.

### 2.1 Surgical procedure and outcome

**Figure 2:**
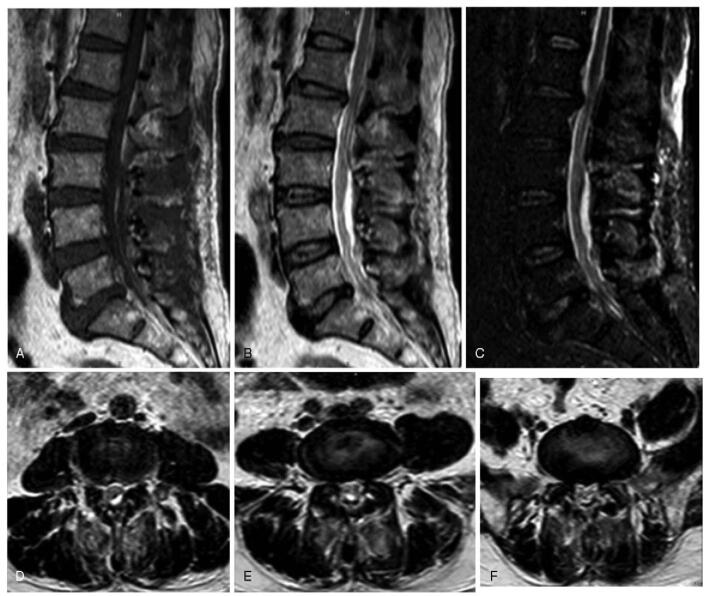
Postoperative MRI showed that the epidural fat had been removed and the dural sac compression was relieved. (A) T1-weighted sagittal image, (B) T2-weighted sagittal image, (C) fat-suppressed T2-weighted image, (D) lumbar axial MRI (L3–L4 level), (E) lumbar axial MRI (L4–L5 level), and (F) lumbar axial MRI (L5–S1 level). MRI = magnetic resonance imaging.

**Figure 3:**
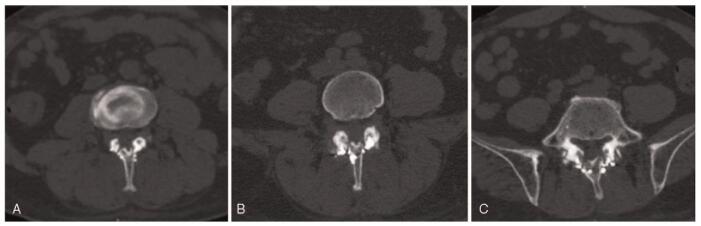
Postoperative computed tomography scan showed that the resected lamina was replanted in situ. (A) L3–L4 level, (B) L4–L5 level, and (C) L5–S1 level.

Under general anesthesia, the patient was placed in the prone position with the abdomen suspended in midair, and the spinous process and lamina (L3–L5) were exposed, and an effort was made to preserve the facet joint capsule. An ultrasonic bone curette was used to resect the spinous process lamina ligament complex (L3–L5). The epidural adipose tissue was removed, and titanium plates and screws were used for spinous process-vertebral plate in situ replantations (Fig. 3). Pathological results showed that the mass was adipose tissue. The patient presented with a complete recovery of radiculopathy and low back pain. Postoperative MRI showed that the increased adipose tissue disappeared and the dural sac compression was relieved (Fig. 2). The 3-month postoperative visual analog scale score score was 0 points and the Oswestry Disability Index was 6.6%. He was able to walk normally, and remained relatively asymptomatic for 12 months after the operation at the last follow-up visit, and a computed tomography scan showed that the replanted laminae were completely healed. This patient provided written informed consent for the publication of the case. This study was approved by the Ethics Committee of the Liaocheng People's Hospital.

## 3 Discussion

SEL was first described by Lee et al (1975).^[7]^ SEL is an overgrowth of epidural adipose tissue in the spinal canal and has been reported to be a rare and particular type of spinal stenosis.^[8,9,10]^ MRI is reported to be the investigative procedure of choice. In a retrospective analytic study by Park et al,^[11]^ lumbar spine MRI showed that the incidence of symptomatic SEL was 1.1%, and SEL was more common at the L5–S1 level and in male patients.

The pathogenesis of SEL remains unclear. According to previous research, long-term exogenous steroid use, epidural corticosteroid injection, metabolic diseases, hypothyroid disease, obesity, and male sex are thought to be risk factors for SEL.^[12,13,14]^ Additionally, approximately 20% of the cases are reported as idiopathic,^[8]^ similar to the current case, associated with unknown reasons. In addition, Greenish et al reported an extremely rare situation, in which a case of acute SEL appeared at the adjacent level directly following bilateral spinal decompression surgery by treatment of lumbar spinal canal stenosis at the L4–L5 level, which indicates that SEL should be recognized as a possible complication of spinal decompression surgery.^[14]^

While SEL can be asymptomatic, surgical intervention should be considered in patients who have failed conservative treatment or in the presence of progressive and severe neurological deficits. Surgical decompression is commonly performed to treat symptomatic SELs. A variety of surgical treatment strategies for SEL have been reported, such as small laminotomy, endoscopically guided fat aspiration, microscopic laminotomy or multilevel laminectomy, fat debulking, and instrumented posterolateral fusion.^[10,15,16,17,18]^

Several studies have demonstrated that extensive laminectomy can induce intervertebral instability and spinal deformities. Although intervertebral fusion and pedicle screw fixation can guarantee the stability of the spine, however, it also brought some relative complications and disadvantages, such as the decreased lumbar mobility, accelerated adjacent segment degeneration, false joint formation,etc.^[19]^

In our case, an MRI of the lumbar spine showed severe spinal canal stenosis at the L3–L4, L4–L5, and L5–S1 levels, due to extensive epidural lipomatosis. Owing to the wide multi-level involvement, and there was no obvious disc herniation or lumbar instability, we performed fat debulking, lamina osteotomy and replantation in situ, and titanium plate fixation for the patient. At present, there are no reports on the surgical treatment of lumbar SEL with laminectomy and in situ replantation. In this way, it not only achieves the purpose of thorough decompression and removal of excess fat from the epidural but also maintains the immediate stability of the spine, preserves lumbar spine mobility, and prevents the formation of spinal scars. In this study, the surgical results were satisfactory during the follow-up.

## 4 Conclusion

At present, there is no uniform consensus on the surgical treatment of symptomatic lumbar SEL, and the surgical approach depends on the specific situation of the patient. The technology of laminotomy and replantation for lumbar SEL can achieve a sufficient and effective decompression, and at the same time, reconstruct the anatomy of the spinal canal. It has the advantages of better maintenance of lumbar stability, preservation of mobility, and reduction of adjacent segment degeneration. Therefore, it is an effective and safe treatment option.

## Acknowledgments

The authors would like to thank the patient for his consent to this case report and for sharing and providing a detailed medical experience.

## Author contributions

**Conceptualization:** Hui Xu.

**Investigation:** Changbin Ji, Dawei Luo.

**Project administration:** Dawei Luo, Kunpeng Li.

**Writing – original draft:** Keshi Yang.

**Writing – review & editing:** Kunpeng Li, Hui Xu.

## References

[1] Bednar DA Esses SI Kucharczyk W. Symptomatic lumbar epidural lipomatosis in a normal male. A unique case report. Spine 1990;15:52–3. 2183369 10.1097/00007632-199001000-00015

[2] Dihlmann SW Mayer HM. Lumbar epidural lipomatosis. Z Rheumatol 1995;54:417–23. 8578893

[3] Bayerl SH Dinkelbach M Heiden P Prinz V Finger T Vajkoczy P. Treatment results for lumbar epidural lipomatosis: does fat matter? Eur Spine J 2019;28:69–77. 30276467 10.1007/s00586-018-5771-1

[4] Min WK Oh CW Jeon IH Kim SY Park BC. Decompression of idiopathic symptomatic epidural lipomatosis of the lumbar spine. Joint Bone Spine 2007;74:488–90. 17681857 10.1016/j.jbspin.2006.11.021

[5] Mallard F Buni M Nolet PS Emary P Taylor JA Moammer G. Lumbar spinal epidural lipomatosis: a case report and review of the literature. Int J Surg Case Rep 2021;78:71–5. 33310475 10.1016/j.ijscr.2020.11.128 PMC7736757

[6] Borré DG Borré GE Aude F Palmieri GN. Lumbosacral epidural lipomatosis: MRI grading. Eur Radiol 2003;13:1709–21. 12835988 10.1007/s00330-002-1716-4

[7] Lee M Lekias J Gubbay SS Hurst PE. Spinal cord compression by extradural fat after renal transplantation. Med J Aust 1975;1:201–3. 1092979 10.5694/j.1326-5377.1975.tb111328.x

[8] Donnarumma P Nigro L Ambrosone A Tarantino R Santoro A Delfini R. Spinal epidural lipomatosis: a rare condition with unclear etiology. J Neurosurg Sci 2019;63:352–4. 29745217 10.23736/S0390-5616.17.04129-7

[9] Sasagasako T Hanakita J Takahashi T Minami M Kanematsu R Tomita Y. Clinical implications of the epidural fat thickness in the management of lumbar spinal stenosis. World Neurosurg 2021;146:e205–13. 33091643 10.1016/j.wneu.2020.10.075

[10] Phalak M Das S Singh M Sharma BS. Unusual cause of lumbar canal stenosis in 8th decade of life – spinal epidural lipomatosis. J Craniovertebr Junction Spine 2017;8:382–3. 29403255 10.4103/jcvjs.JCVJS_103_15 PMC5763600

[11] Park SK Han JM Lee K Cho WJ Oh JH Choi YS. The clinical characteristics of spinal epidural lipomatosis in the lumbar spine. Anesth Pain Med 2018;8:e83069. 30538942 10.5812/aapm.83069 PMC6252047

[12] Moller JC Cron RQ Young DW. Corticosteroid-induced spinal epidural lipomatosis in the pediatric age group: report of a new case and updated analysis of the literature. Pediatr Rheumatol Online J 2011;9:05. 10.1186/1546-0096-9-5 PMC3041993 21284882

[13] Yoo JC Choi JJ Lee DW Lee SP. Spinal epidural lipomatosis in korean. J Korean Neurosurg Soc 2014;55:365–9. 25237435 10.3340/jkns.2014.55.6.365 PMC4166335

[14] Greenish D Watura K Harding I. Spinal epidural lipomatosis following bilateral spinal decompression surgery. BMJ Case Rep 2019;12:e226985. 10.1136/bcr-2018-226985 PMC6388899 30772832

[15] Lisai P Doria C Crissantu L Meloni GB Conti M Achene A. Cauda equina syndrome secondary to idiopathic spinal epidural lipomatosis. Spine 2001;26:307–9. 11224868 10.1097/00007632-200102010-00017

[16] Sairyo K Sakai T Higashino K Hirao B Katoh S Yasui N. Minimally invasive excision of lumbar epidural lipomatosis using a spinal endoscope. Minim Invasive Neurosurg 2008;51:43–6. 18306131 10.1055/s-2007-1004569

[17] Frank E. Endoscopic suction decompression of idiopathic epidural lipomatosis. Surg Neurol 1998;50:333–5. 9817455 10.1016/s0090-3019(98)00016-0

[18] Ferlic PW Mannion AF Jeszenszky D. Patient-reported outcome of surgical treatment for lumbar spinal epidural lipomatosis. Spine J 2016;16:1333–41. 27363757 10.1016/j.spinee.2016.06.022

[19] Hao X Lin W. Vertebral plate and ligament composite laminoplasty in spinal cord tumor surgery: analysis of 94 patients. Transl Neurosci 2021;12:40–5. 33552593 10.1515/tnsci-2021-0007 PMC7821417

